# Transcranial ultrasound pulse stimulation reduces cortical atrophy in Alzheimer's patients: A follow‐up study

**DOI:** 10.1002/trc2.12121

**Published:** 2021-02-25

**Authors:** Tudor Popescu, Cyril Pernet, Roland Beisteiner

**Affiliations:** ^1^ Department of Behavioural and Cognitive Biology University of Vienna Vienna Austria; ^2^ Department of Neurology Medical University of Vienna Vienna Austria; ^3^ Centre for Clinical Brain Sciences University of Edinburgh Edinburgh UK

**Keywords:** Alzheimer's disease, brain stimulation, cortical atrophy, cortical thickness, default mode network, ultrasound

## Abstract

**Introduction:**

Ultrasound for the brain is a revolutionary therapeutic concept. The first clinical data indicate that 2–4 weeks of therapy with transcranial pulse stimulation (TPS) improve functional networks and cognitive performance of Alzheimer's disease (AD) patients for up to 3 months. No data currently exist on possible benefits concerning brain morphology, namely the cortical atrophy characteristic of AD.

**Methods:**

We performed a pre‐/post‐therapy analysis of cortical thickness in a group of N = 17 AD patients.

**Results:**

We found a significant correlation between neuropsychological improvement and cortical thickness increase in AD‐critical brain areas.

**Discussion:**

AD patients who benefit from TPS appear to manifest reduced cortical atrophy within the default mode network in particular, whose memory‐related subsystems are believed to be disrupted in AD. TPS may therefore hold promise as a new add‐on therapy for AD.

## 1 INTRODUCTION

Ultrasound for the brain is a revolutionary new therapeutic concept. Transcranial techniques such as high‐intensity focused ultrasound are already in clinical use for non‐invasive focal surgery, and clinical feasibility studies exist for blood‐brain barrier opening for targeted local drug applications or local gene therapy.^1^, ^2^, ^3^, ^4^ Very recently, a first clinical study has presented a third major modality of ultrasound application: therapeutic brain activation in Alzheimer's disease (AD).^2^ This study applied a novel pulsed ultrasound technique, transcranial pulse stimulation (TPS).^3^ There were two major outcomes: (1) memory improvements up to 3 months post‐stimulation, and (2) neurophysiological functional magnetic resonance imaging (fMRI) and electroencephalogram (EEG) evidence for improved brain activity and memory network connectivity. TPS is particularly promising, because it represents an add‐on therapy that may be applied concurrently with established therapies and therefore increase patients' chance of improvement. In pathological brains, in which tissue conductivity can be profoundly altered, electromagnetic methods of transcranial non‐invasive brain stimulation (such as transcranial magnetic‐ and direct current stimulation, TMS and tDCS) are difficult to target.^6^ In contrast, ultrasound stimulation can be targeted exactly in such cases, because ultrasound does not depend on intracerebral conductivities.^4^ Further ultrasound stimulation and TPS allow stimulation of deep subcortical areas.^5^ As known from a large body of literature, neuroplastic rehabilitation involves not only functional^7^ but also cortico‐morphological changes, in terms of, for instance, MR‐derived cortical thickness or voxel‐based morphometry.^8^, ^9^ However, currently no human or animal data exist on whether therapy based on ultrasound brain stimulation can also lead to morphological (namely, cortical thickness) changes. Here, we aim to fill this gap. Based on the recent evidence of functional improvement in AD patients, we investigate a possible effect of ultrasound stimulation (in the form of TPS) on cortical thickness in those same patients.

## 2 METHODS

### 2.1 Participants

We analyzed data from the 20 AD patients of our previous TPS study,^2^ for whom structural MR data (T1‐weighted, magnetization‐prepared rapid acquisition with gradient echo [MPRAGE] sequence) were available. The post‐scans of two of the patients, and the pre‐scan of one patient, were not available, leaving a sample size of N = 17 with complete pre–post MR data. The quality of cohort was generally “mild AD,” with clinical AD classification according to the National Institute on Aging‐Alzheimer's Association criteria.

The study inclusion/exclusion criteria were rather broad, to reflect the real out‐patients’ situation. Namely, inclusion criteria were: clinically stable patients with probable AD, at least 3 months of stable antidementia therapy (if any), signed informed consent, age ≥18 years. Exclusion criteria were: noncompliance with the protocol, relevant intracerebral pathology unrelated to the AD (eg, brain tumor), hemophilia or other blood clotting disorders or thrombosis, corticosteroid treatment within the last 6 weeks before first treatment.

The protocol for the study has received prior approval by the appropriate institutional review board (MUV 1227/2015). Informed consent was obtained from each patient.

### 2.2 Procedure

Most patients received TPS therapy for 4 weeks (three patients for only 2 weeks, one for 3 weeks). Functional and morphological MR data were recorded before and after TPS therapy.

**Highlights**

- Ultrasound for the brain provides revolutionary and effective therapeutic concepts.
- Transcranial pulse stimulation (TPS) can precisely target cortical and subcortical areas.
- TPS has been shown to improve functional networks and cognitive performance in Alzheimer's disease (AD)
- We report such pre–post TPS changes that are predictive of cortical thickness increase.
- TPS might reduce cortical atrophy within AD‐critical brain areas such as the default mode network.

**Research in context**

1. Systematic review: Our previous study indicates that therapy with ultrasound brain stimulation improves functional networks and cognitive performance of Alzheimer's disease (AD) patients, long term. These prior results informed our selection of regions of interest for the present study. We found no other published clinical studies on the brain‐functional and ‐morphological correlates of ultrasound brain stimulation; this was ascertained by searching PubMed using the terms (“ultrasound” AND “brain stimulation”) AND (“magnetic resonance” OR “neuroimaging”) AND (“clinical” OR “Alzheimer's”).
2. Interpretation: As patients improve cognitively, cortical thickness—in AD‐relevant brain regions—increases. We demonstrate, for the first time, that therapeutic ultrasound may change brain morphology, possibly by reducing cortical atrophy.
3. Future directions: These and other (preclinical) results provide ample evidence that ultrasound for the brain is an effective therapeutic concept for precisely targeted non‐invasive brain stimulation. Ultrasound may therefore act as an add‐on therapy for AD by inducing therapeutically relevant functional and morphological changes.

#### 2.2.1 TPS therapy and region of interest selection

Individual regions of interest (ROIs) were defined by a neurologist (R.B.) to target AD‐relevant brain areas, that is, the AD network, consisting of: the classical AD stimulation target dorsolateral prefrontal cortex, areas of the memory (including default mode) and language networks. Specifically, ROIs comprised: bilateral frontal cortex (dorsolateral prefrontal cortex and inferior frontal cortex extending to Broca's area, ROI volume 136/164 cm³ – 2 × 800 pulses per hemisphere), bilateral lateral parietal cortex (extending to Wernicke's area, ROI volume 122/147 cm³ – 2 × 400 pulses per hemisphere), and extended precuneus cortex (1 bilateral volume with 66/92 cm³ – 2 × 600 pulses). The goal was to distribute all pulses within the respective ROIs with a focus on the cortical tissue. Every ROI was stimulated twice per session.

Standardized target volumes of interest (ellipsoidal ROIs) were defined for each individual participant based on their structural MRI. This Individual real‐time tracking enabled standardized focal brain stimulation over the whole study population with adequate movements of the hand piece over the skull.

A NEUROLITH TPS generator (Storz Medical AG, Tägerwilen, Switzerland) was used, with single ultrasound pressure pulses: duration about 3 μs, 0.2 mJ mm^−2^ energy flux density, pulse repetition frequency 5 Hz, pulse number per therapeutic session 6000. All regulations required by the Conformité Européenne (CE) authorities have been fulfilled for acquisition of the CE mark for TPS.

#### 2.2.2 MRI sequence parameters

A T1‐weighted structural image was recorded using a MPRAGE sequence (TE/TR = 2.7/1800 ms, inversion time = 900 ms, flip angle = 9°, resolution 1 mm isotropic).

#### 2.2.3 Neuropsychological testing

Neuropsychological evaluation was done with the German version of the CERAD Plus (Consortium to Establish a Registry for Alzheimer's Disease) test battery,^10^ which included tests for: word fluency (phonemic and categorical), naming (Boston Naming Test), encoding, recognition, and recall of verbal material (Word List), as well as constructional praxis and constructional recall (Figures Copy and Recall). CERAD raw scores were used to calculate a corrected total score (CTS) for each patient. CERAD does not suffer from repetition effects in mild AD.^11^, ^12^

### 2.3 Analyses

#### 2.3.1 Cortical thickness derivation and analysis

We used a surface‐based morphometry approach to analyze our anatomical images, as implemented in FreeSurfer (Massachusetts General Hospital, Harvard University, Cambridge, Massachusetts, USA). This involves a standard pre‐processing stream to derive cortical surface models (meshes), followed by a longitudinal processing stream to produce cortical thickness values at both time points; details of these procedures can be found elsewhere.^13^, ^14^, ^15^, ^16^, ^17^

For the group analysis of cortical thickness, a simple two‐stage model was used, which performs a linear fit in each subject independently, to reduce the repeated measures to a single slope, and then performs a regular cross‐sectional analysis across subjects, to look for a pre–post effect.^15^, ^16^

#### 2.3.2 Regions of interest and correlational approach

Our previous fMRI data showed that the entorhinal, parahippocampal, lateral parietal (inferior and superior), and precuneus areas responded most significantly to TPS therapy, in the sense that CTS increase was predicted by activation and connectivity of these areas.^2^ Therefore, to examine the case of morphology, we computed correlations between pre‐to‐post changes in CERAD CTS and pre‐to‐post changes in the cortical thickness of these areas, as defined in the Desikan‐Killiany atlas.^14^ Both robust correlations,^18^ that is, skipped Pearson's *r* that accounts for bivariate outliers, and multiple testing correction^19^ were used, ensuring valid inference.^20^

### 2.4 Data availability

The datasets generated during and/or analyzed during the current study are available in the *TPS_CorticalThickness_Alzheimers* repository, at osf.io/4gsj5.

## 3 RESULTS

### 3.1 No pre‐to‐post change at group level

While a significant pre‐to‐post behavioral improvement in CTS score was observed in this N = 17 subgroup (3.76 ± 5.35, *t*[16] = 2.89, *P *= .01), the pre‐to‐post group‐level difference at the whole brain level produced no significant clusters after family‐wise error (FWE) correction for multiple comparisons.

### 3.2 Memory areas that respond functionally to TPS also respond morphologically

**FIGURE 1:**
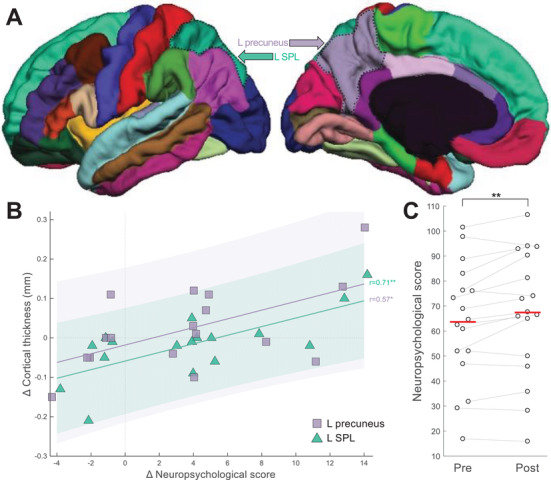
**Brain–behavior correlations**. **A**, Regions of interest—namely left superior parietal lobule (SPL) and left precuneus—where a significant correlation (plotted in panel [**B**]) was observed between pre‐to‐post change in cortical thickness and in neuropsychological scores (corrected total score [CTS]). The two regions of interest are highlighted with dotted black contours on lateral and medial views of the DKT atlas parcellation; base image from the Mindboggle project (www.dataverse.harvard.edu/dataverse/mindboggle101). **B**, Data points in the scatter plot represent patients (N = 17; SPL: green triangles; precuneus: purple squares). Data points sharing the same x value are horizontally jittered by up to 0.25 units to aid visualization. Vertical/horizontal dotted gray lines correspond to no pre‐to‐post change in terms of neuropsychological score/cortical thickness. “Δ” in the axis labels refers to pre‐to‐post‐TPS change. **C**, Distribution of neuropsychological (CTS) scores, pre‐ and post‐TPS. Dots represent individual patients, with gray lines connecting values at pre and at post. Horizontal red lines indicate group means. ^*^ *P *< .05; ^**^ *P *< .01

Focusing on the regions of interest suggested by our previous study, we found significant correlations between changes (pre‐to‐post TPS) in the neuropsychological CTS score and in the cortical thickness of structures of the AD‐critical default mode network, namely of the left superior parietal lobule (*r *= 0.70 [0.40 0.85], *P *= .0017) and left precuneus (*r *= 0.39 [0.10 0.80], *P *= .03), with only the left superior parietal lobule being significant after correcting for multiple testing (Figure 1, Table 1). Adjusting gray matter thickness for age, the results remain unchanged (left superior parietal: *r *= 0.69 [0.40 0.80], *P *= .0016; and left precuneus: *r *= 0.56 [0.19 0.80], *P *= .0160).

**TABLE 1:** Brain–behavior correlations

ROI	Pearson's *r*	95% CI for *r*	*t*	df	*P*
Right entorhinal	0.3047	[−0.1999, 0.7024]	1.2391	15	.2304
Left enthorinal	0.0075	[−0.3617, 0.3678]	0.0290	15	.9082
Right inferior parietal	0.4184	[−0.1388, 0.7372]	1.7840	15	.1703
Left inferior parietal	0.3470	[−0.2042, 0.6718]	1.4328	15	.2237
Right parahippocampal	0.3936	[−0.1830, 0.7523]	1.6581	15	.1569
Left parahippocampal	0.3630	[−0.1071, 0.6865]	1.5086	15	.0968
Right precuneus	0.4683	[−0.0865, 0.7828]	2.0526	15	.0768
Left precuneus	0.3941	[0.1008, 0.8466]	1.6607	15	.0301
Right superior parietal	0.0906	[−0.2952, 0.4440]	0.3525	15	.6411
Left superior parietal	0.7018	[0.4020, 0.8567]	3.8156	15	**.0017**

Notes: Robust Pearson correlations between pre‐to‐post‐TPS change in neuropsychological score (CTS) and pre‐to‐post‐TPS change in cortical thickness of all the functionally relevant ROIs considered. The *P*‐value in bold (left superior parietal) is significant also after correcting for multiple comparisons using a projection‐type outlier detection method.^19^

Abbreviations: CI, confidence interval; CTS, corrected total score; ROI, region of interest; TPS, transcranial pulse stimulation.

## 4 DISCUSSION

Our study provides the very first data concerning ultrasound brain stimulation and brain morphology in humans. Results indicate that the revolutionary concept of ultrasound as an additional avenue for neurodegeneration therapy, may modulate cortical thickness of stimulated brain areas and thereby reduce cortical atrophy in AD. This was found in a set of regions known to be particularly important for AD patients' cognitive functioning—the default mode network. This network is important for cognitive and memory performance and degenerates very early in the course of AD. Importantly, a global cortical thickness effect—extending to brain areas less relevant for AD—was absent. These data confirm expectations^7^ that therapeutic ultrasound may not only have functional effects—something demonstrated in a large variety of animal and human studies—but may also reduce atrophy of brain tissue that is treated with TPS. It is important to note that the principle of therapeutic neuromodulation with TPS that we describe here need not only apply to AD but, plausibly, to all neuropsychiatric diseases for which neuroplastic reorganization may be helpful. If this is true, then in the case of AD and its specific neuropathology, our results could be incipient evidence that TPS also acts by reducing cortical atrophy.

The exact cellular mechanisms behind ultrasound‐based stimulation, and the direction of the overall effects, that is, whether they cause an increase or decrease in excitability at a systems level, is currently under intense research. Most likely, ultrasound neuromodulation rests on an initial change of cell membrane permeability.^21^ This causes a cascade of transmitter, humoral factor, and cell activity changes with long‐term neuroplastic effects. In our previous clinical AD study with TPS, the procedure was well tolerated and after treatment, memory performance improved for up to 3 months. These results parallel those of another brain stimulation study investigating the long‐term effects of TMS as a treatment for AD.^22^

These are the very first in vivo data on ultrasound related brain‐morphological changes, however several limitations of the study have to be considered. First, TPS stimulation has not been controlled with a *sham* stimulation. Notwithstanding, our previous neuropsychological and functional data demonstrated that only stimulated brain networks improved, while non‐stimulated networks worsened, in that the corresponding neuropsychological performance deteriorated—according to the expected course of the disease.^2^ In addition, the long‐term course of neuropsychological improvements clearly differed from the known placebo responses.^23^ This corresponds well to our present finding of no global cortical thickness effect, but of specific effects in functionally relevant areas. Second, with this pilot study MR data are only available for pre‐/post‐therapy time points spanning 2–4 weeks. Whether morphological changes may be induced for longer time periods—comparable to the neuropsychological 3‐months‐on effects—remains to be investigated.

In conclusion, available evidence indicates that therapeutic ultrasound with TPS may indeed be effective as a new add‐on therapy for AD and possibly for other neurodegenerative diseases. Besides inducing memory improvements, here an associated reduction of cortical atrophy is suggested. This result remains to be confirmed or refuted in future clinical trials that can provide higher quality of evidence.

## CONFLICTS OF INTEREST

The authors declare no competing interests.

## AUTHOR CONTRIBUTIONS

Tudor Popescu analyzed the data, generated the figures, and wrote the manuscript. Cyril Pernet helped with study design and data analyses. Roland Beisteiner designed the study, recruited patients, gave input in data collection, and wrote parts of the manuscript. All authors approved the final contents of the manuscript.

## Supporting information

Supplementary information

Supplementary information
