# Supplementary material for: Transcranial ultrasound pulse stimulation reduces cortical atrophy in Alzheimer's patients: A follow‐up study
Source: Alzheimers Dement (N Y). 2021 Feb 25;7(1):e12121. doi: 10.1002/trc2.12121 (PMC7906128; doi:10.1002/trc2.12121)
Supplement: Supplementary file 1 — Supplementary information [file TRC2-7-e12121-s001.docx]

**Systematic review:** Our previous study indicates that therapy with ultrasound brain stimulation improves functional networks and cognitive performance of AD patients, long-term. These prior results informed our selection of regions of interest for the present study. We found no other published clinical studies on the brain-functional and -morphological correlates of ultrasound brain stimulation; this was ascertained by searching PubMed using the terms ("ultrasound" AND "brain stimulation") AND ("magnetic resonance " OR "neuroimaging") AND ("clinical" OR "Alzheimer's").

**Interpretation:** As patients improve cognitively, cortical thickness – in AD-relevant brain regions – increases. We demonstrate, for the first time, that therapeutic ultrasound may change brain morphology, possibly by reducing cortical atrophy.

**Future directions:** These and other (preclinical) results provide ample evidence that ultrasound for the brain is an effective therapeutic concept for precisely targeted non-invasive brain stimulation. Ultrasound may therefore act as an add-on therapy for AD by inducing therapeutically relevant functional and morphological changes.
