# Supplementary material for: Transcranial ultrasound pulse stimulation reduces cortical atrophy in Alzheimer's patients: A follow‐up study
Source: Alzheimers Dement (N Y). 2021 Feb 25;7(1):e12121. doi: 10.1002/trc2.12121 (PMC7906128; doi:10.1002/trc2.12121)
Supplement: Supplementary file 2 — Supplementary information [file TRC2-7-e12121-s002.docx]

- Ultrasound for the brain provides revolutionary and effective therapeutic concepts
- Transcranial Pulse Stimulation (TPS) can precisely target cortical&subcortical areas
- TPS has been shown to improve functional networks and cognitive performance in AD
- We report such pre-post-TPS changes being predictive of cortical thickness increase
- AD patients might reduce cortical atrophy within AD-critical brain areas e.g. DMN
